# *De Novo* Assembly of the Pea (*Pisum sativum* L.) Nodule Transcriptome

**DOI:** 10.1155/2015/695947

**Published:** 2015-11-24

**Authors:** Vladimir A. Zhukov, Alexander I. Zhernakov, Olga A. Kulaeva, Nikita I. Ershov, Alexey Y. Borisov, Igor A. Tikhonovich

**Affiliations:** ^1^All-Russia Research Institute for Agricultural Microbiology (ARRIAM), Podbelsky Chausse 3, Saint Petersburg 196608, Russia; ^2^Institute of Cytology & Genetics SB RAS, Prospekt Lavrentyeva 10, Novosibirsk 630090, Russia; ^3^Saint Petersburg State University, Universitetskaya Embankment 7-9, Saint Petersburg 199034, Russia

## Abstract

The large size and complexity of the garden pea (*Pisum sativum* L.) genome hamper its sequencing and the discovery of pea gene resources. Although transcriptome sequencing provides extensive information about expressed genes, some tissue-specific transcripts can only be identified from particular organs under appropriate conditions. In this study, we performed RNA sequencing of polyadenylated transcripts from young pea nodules and root tips on an Illumina GAIIx system, followed by* de novo* transcriptome assembly using the Trinity program. We obtained more than 58,000 and 37,000 contigs from “Nodules” and “Root Tips” assemblies, respectively. The quality of the assemblies was assessed by comparison with pea expressed sequence tags and transcriptome sequencing project data available from NCBI website. The “Nodules” assembly was compared with the “Root Tips” assembly and with pea transcriptome sequencing data from projects indicating tissue specificity. As a result, approximately 13,000 nodule-specific contigs were found and annotated by alignment to known plant protein-coding sequences and by Gene Ontology searching. Of these, 581 sequences were found to possess full CDSs and could thus be considered as novel nodule-specific transcripts of pea. The information about pea nodule-specific gene sequences can be applied for gene-based markers creation, polymorphism studies, and real-time PCR.

## 1. Introduction

Pea (*Pisum sativum* L.), an important crop cultivated worldwide [1], is a valuable model system in plant genetics. Since Gregor Mendel's famous experiments, several scientific discoveries have occurred in modern pea genetics; these new insights include information regarding genetic control of compound leaf development [2, 3] and the molecular basis of symbiotic interactions with beneficial nitrogen-fixing bacteria (rhizobia) [4–6]. The study of pea gene polymorphism in relation to agronomically important traits is essential to both basic and applied research on this crop plant [7, 8]. Unfortunately, the large size (more than 4 Gb) (http://data.kew.org/cvalues/) and complexity [9] of the pea genome hamper its sequencing as well as the discovery of this crop plant's genetic resources, both of which are desperately needed for molecular and genomics-assisted breeding [8, 10].

As an alternative to whole genome sequencing, analysis of transcriptomes by RNA sequencing can provide extensive information about expressed genes [11, 12]. Because next-generation sequencing technologies are applicable to all organisms, including those for which information about genome organization is insufficient or lacking, considerable progress in pea transcriptome sequencing has been achieved over the last few years. Massive amounts of transcriptomic data have been obtained in the form of high-quality sequence reads that have been used for molecular marker creation, whole genome map construction [13–17], and characterization of host-pathogen interactions (pea-*Sclerotinia sclerotiorum*) [18]. All these data (as well as additional unpublished pea transcriptome sequencing results) have been uploaded to the NCBI Sequence Read Archives (SRA) (http://www.ncbi.nlm.nih.gov/sra/) as raw reads. Assemblies have been created for some of these data and deposited in the NCBI Transcriptome Shotgun Assembly (TSA) database, allowing users to perform data mining (e.g., BLAST searching) and to study pea gene polymorphism.

Several genes have tissue-specific expression, however, and can therefore only be studied through analysis of the appropriate tissue. One such example involves symbiotic genes necessary for the establishment and development of nitrogen-fixing nodules which are predominantly expressed in those temporary plant organs. To date, only a few samples from pea nodules have been sequenced (available as raw SRA archives), and only one assembly built from a mixture of sequencing reads from different organs (including nodules) is present in the TSA database (see Table 1 for available pea nodule transcriptome sequencing results). Because this assembly was based on nodules harvested at a very late stage of symbiotic nodule development (3-month-old plants), it presumably contains insufficient information on nodule-specific transcripts. Consequently, sequences of nodule-specific genes of pea are still limited.

The aim of our work was thus to sequence the transcriptome of young pea nodules, construct an assembly, and analyze the resulting assembly for unique sequences. Along with nodules, we harvested root tips to analyze their transcriptome content as well.

## 2. Materials and Methods

### 2.1. Biological Materials

Seeds of pea laboratory line SGE [19] were surface-sterilized with concentrated sulfuric acid (98%) (15 min on a shaker), washed 10 times with autoclaved distilled water, and germinated on Petri dishes containing sterile vermiculite for 3 days. The germinated seeds were then planted individually into 200 mL ceramic pots containing quartz sand, watered with 100 mL of 2x nitrogen-free mineral nutrition solution [20], and inoculated with an aqueous suspension of* Rhizobium leguminosarum* bv.* viciae* RCAM1026 [21] (1 × 10^6^ CFU per plant). Plants were harvested 12 days after inoculation; nodules and root tips (5 mm distal portion of the root) were placed in liquid nitrogen, ground into powder, and stored at −80°C. Material was harvested from a total of 10 plants.

### 2.2. cDNA Library Construction and Sequencing

Total RNA was extracted from 100 *μ*g of material using an RNeasy Plant mini kit (Qiagen, Hilden, Germany). cDNA libraries were constructed and sequenced according to the instructions provided with the Genome Analyzer IIx platform (Illumina, San Diego, CA, USA). After total RNA extraction and DNase-I treatment, mRNAs were captured using oligo (dT) magnetic beads and fragmented. First-strand cDNA was synthesized from these fragments using random hexamer primers; double-stranded cDNA was then generated, purified with magnetic beads, and subjected to end reparation and 3′ single adenylation. Sequencing adaptors were ligated to the adenylated fragments, and DNA fragments having adapter molecules on both ends were then amplified. After a quality control step performed on a 2100 Bioanalyzer (Agilent Technologies, Santa Clara, CA, USA), the cDNA library products were sequenced in a single-read run with 75 bp length reads on an Illumina Genome Analyzer IIx platform.

### 2.3. *De Novo* Transcriptome Assembly

Preliminary quality control of the raw sequencing data was performed via the FastQC v.0.11.3 application (http://www.bioinformatics.babraham.ac.uk/projects/fastqc/), which indicated that the reads were of acceptable quality. For adapter removing Cutadapt version 1.8.1 [22] was used. Low-quality read removal and trimming were then performed with the assembly program Trinity v.2.0.6 [23] using the “trimmomatic” option with default parameters. Next, contig assembly was performed by Trinity with default assembly parameters, including kmer = 25. As a result, two FASTA files were obtained, one for the nodule sample and one for the root tip sample. Statistical parameters for the assemblies were obtained by running the TrinityStats.pl script included in the Trinity package.

### 2.4. Assessment of Assembly Quality, Differential Expression Analysis, and Functional Annotation of Contigs

As a step in assessment of assembly quality, the generated reads were mapped to the assemblies with the Bowtie2 program v. 2.2.5 [24]. The contigs were grouped with known pea sequences (obtained from http://www.ncbi.nlm.nih.gov/) using CD-HIT_EST from the CD-HIT package (http://cd-hit.org/) [25] with parameters -c 0.80 -n 6.

In order to distinguish the transcripts enriched in nodules as compared to root tips, the reads of both libraries were mapped to “Nodules” assembly with Bowtie2 v. 2.2.5 [24]. The differential expression was calculated using EdgeR package [26] under a negative binomial model, with biological coefficient of variation 0.2 and FDR cutoff value 0.001.

The assemblies were compared to the NCBI nonredundant (nr) database using BLASTX [27]. The resulting BLAST output was processed using publicly available Blast2GO software (v.2.5.0) (BioBam Bioinformatics SL, Valencia, Spain) [28] to retrieve associated Gene Ontology (GO) terms describing biological processes, molecular functions, and cellular components [29].

To detect transcripts containing reliable full-length CDS regions two approaches based on similarities of either nucleotide or amino acid sequences were used. For each transcript, BLAST search against NCBI RefSeqGene database was performed in order to find orthologous sequences, and then these sequences were aligned by Smith-Waterman algorithm [30] with a “5-0” substitution matrix. Also, as an alternative approach, we used TransDecoder software [23] for CDS region prediction based on homology search against Swiss-Prot protein database [31].

To extend the annotation of the full-length nodule-specific transcripts, the nucleotide sequences were converted into amino acid sequences and then mapped to the Kyoto Encyclopedia of Genes and Genomes (KEGG) Web Server (http://www.genome.jp/kegg/) [32].

### 2.5. Sanger Sequencing, Primer Design, and Online Computational Tools

Direct sequencing of PCR fragments was performed on an ABI Prism 3500 xL system (Applied Biosystems, USA) at the Genomic Technologies, Proteomics, and Cell Biology Core Center of All-Russia Research Institute for Agricultural Microbiology (ARRIAM, Saint Petersburg, Russia). The online tool OligoCalc [33] was used for primer design. Alignments of small sequence sets were generated using Multalin [34]. Translation initiation site prediction was performed using NetStart 1.0 [35].

## 3. Results and Discussion

### 3.1. Sequencing and Assembly

**Figure 1:**
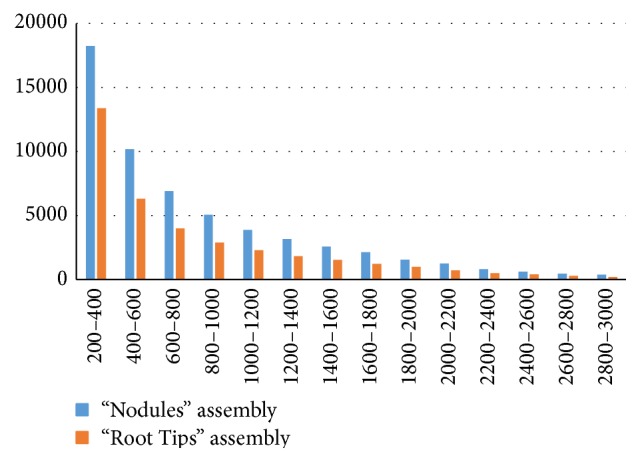
Length distribution of contigs obtained from “Nodules” and “Root Tips” assemblies.

**Figure 2:**
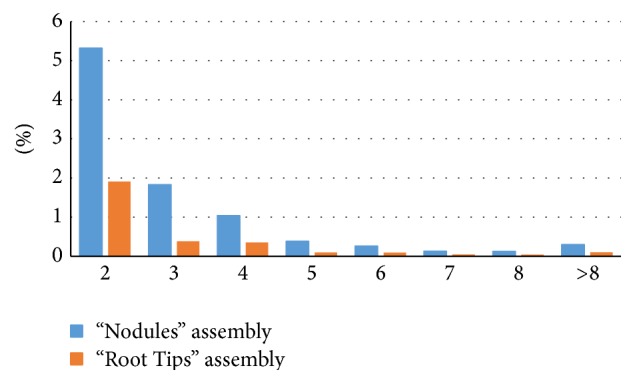
Isoform distribution in “Nodules” and “Root Tips” assemblies.

Sequencing generated 52,021,865 reads from the “Nodules” library and 17,684,604 reads from the “Root Tips” library. After removal of adapter and index sequences, 75.1% reads were equal to or longer than 70 bp and 10.2% of reads were shorter than 10 bp for “Nodules” and 74.5% and 11.2% of reads, accordingly, for “Root Tips.” The nodule and root tip read sets were assembled individually. A total of 58,397 contigs belonging to 48,628 genes (as termed by Trinity) were constructed from the nodule set, with a mean contig length of 880.81, a median contig length of 620, and an N50 of 1,282. Isoforms were proposed for 4,550 genes. Root tip reads were assembled into 37,287 contigs of 35,081 genes, with a mean contig length of 841.14, a median contig length of 558, an N50 of 1,260, and 1,055 total isoforms. Length distributions of contigs in the two assemblies are presented in Figure 1, and distributions of isoform numbers are shown in Figure 2.

To our knowledge, the appearance of isoforms can be due to either alternative splicing or the presence of paralogous sequences expressed in the tissue. As an illustration of the first case, we were able to detect two splice variants in the “Nodules” assembly for transcripts of the symbiotic gene* Ign1*, an ortholog of* IGN1* (*Ineffective Greenish Nodules 1*) of* Lotus japonicus* (Regel.) K. Larsen [36] (GenBank accession number KR047192; TR2831|c0_g2_i1 and TR2831|c0_g2_i4 in the “Nodules” assembly). The longer transcript retained the first intron and, according to prediction by NetStart 1.0 [35], could be translated into a protein variant lacking the first 21 N-terminal amino acids. An example of the second case involved* ENOD6* (for early nodulin 6; GenBank accession number X63700), which encodes a short protein belonging to a group of nodule-specific cysteine-rich (NCR) peptides [37, 38]. BLASTN searching uncovered a group of isoforms (TR2035 from the “Nodules” assembly) derived from paralogous genes encoding cysteine-rich peptides specific for nodules (it should be noted that some of these isoforms could be artificial chimeras containing parts of different transcripts that have extensive segments sharing 100% similarity).

### 3.2. Quality Assessment

Evaluating the quality of a* de novo* transcriptome assembly without a reference genome is challenging. We therefore implemented three approaches previously recommended for managing this task [39, 40].

First, we used the pea expressed-sequence tag (EST) sequences represented in GenBank as a standard to estimate assembly quality. We aligned 18,576 ESTs against the “Nodules” assembly. Of these, 2,571 ESTs (13.8%) shared no similarity with any contigs of the assembly. Furthermore, 102 ESTs were filtered out on the basis of an* E*-value cutoff of 1 × 10^−10^. From the remaining 15,903 ESTs (85.6%), we chose hits with maximal coverage of the EST (one per EST) and evaluated their coverage and identity distributions. Among these EST-contig pairs, 94.9% of the ESTs shared more than 90% identity with their corresponding contig fragments.

Following the second recommended approach, we mapped back all reads to contigs in both assemblies; as a result, 89% and 91% of reads in “Nodules” and “Root Tips” assemblies were, respectively, aligned back to the contigs, demonstrating that our assemblies were of acceptable quality.

Third, being interested in symbiosis-specific genes, we searched the “Nodules” assembly for previously unknown pea homologs of symbiotic genes* EFD* (ethylene response factor required for nodule differentiation) [41],* VPY* (Vapyrin) [42], and* NSP1* (nodulation signaling pathway 1) [43] of* Medicago truncatula* Gaertn. and* SEN1* (stationary endosymbiont nodule 1) [44] of* L. japonicus*. Long transcripts with high identity were found for all four genes (Table 2); these transcripts allowed us to design primers flanking coding sequence (CDS) regions and to amplify the corresponding regions in cDNA synthesized from 4-week-old nodules of pea genotypes SGE and Finale. Except for allelic variations of* Vpy*,* Sen1*, and* Nsp1* that were found between SGE transcriptome and Finale cDNA genotypes, we observed complete sequence correspondence for all four genes, thereby demonstrating the satisfactory quality of the created assembly.

To evaluate the “Root Tips” assembly, we selected genes involved in glutathione biosynthesis:* Gsh1* (gamma-glutamylcysteine synthetase precursor [AF128455.1]),* Gshs* (glutathione synthetase precursor [AF231137.1]), and* hGshs* (putative homoglutathione synthetase [AF258319.1]). BLASTN searching against the “Root Tips” assembly identified one contig (TR9283|c0_g1_i1) completely identical to full-length* Gsh1* and one contig (TR8244|c0_g1_i1) completely identical to full-length* Gshs* except for a single nucleotide polymorphism in the 3′ untranslated region. This search also revealed four contigs (TR2244|c0_g1_i1, TR3920|c0_g1_i1, TR13401|c0_g1_i1, and TR25033|c0_g1_i1) representing portions of* hGshs* (each with 100% identity) that had not been assembled into a contig, probably because of insufficient overlapping of reads due to the low expression levels of this gene in pea root tips. Consequently, despite the good quality of the “Root Tips” assembly, its coverage was insufficient for finding full sequences of rare transcripts; nevertheless, the discovery of partial sequences allows primers to be designed for whole-transcript PCR amplification and transcript-end amplification by rapid amplification of cDNA ends (RACE) methodology.

### 3.3. Annotation of Nodule-Specific Transcripts

**Figure 3:**
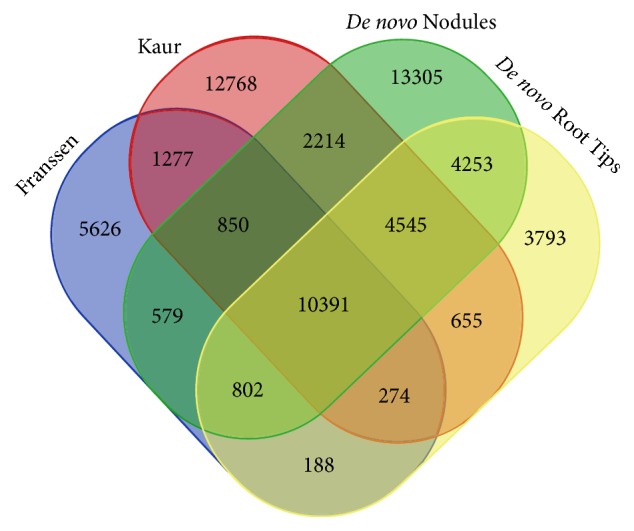
Clustering the sequences of the “*de novo* Nodules” assembly (this study) together with pea sequences produced from nonnodular tissues (“Franssen” [13], “Kaur” [14], and “*de novo* Root Tips” (this study)).

To obtain information on nodule-specific genes, we attempted to select portions of previously unknown sequences of the “Nodules” assembly by clustering them together with pea sequences produced from nonnodular tissues. In addition to our “Root Tips” assembly, the pea transcriptome assemblies created by Franssen et al. [13] and Kaur et al. [14] currently meet this requirement. Using Cd-Hit software, 277,211 sequences of these four pea transcriptome assemblies were grouped into 61,521 clusters (where a cluster is defined according to Cd-Hit as a set of similar sequences created to reduce sequence redundancy and to improve the performance of other sequence analyses). Among these clusters, 10,391 (approximately 17%) were common to all assemblies (Figure 3). The 13,305 nodule-specific clusters included 14,998 contigs belonging to 14,171 genes (i.e., without gene isoforms). These sequences were assigned to GO terms to characterize the nodule transcriptome profile.

The transcripts were first aligned against plant protein sequences in the NCBI nr protein database (24.04.15 release). The following parameters were used: an* E*-value cutoff of 1 × 10^−20^, the same alignment direction for all high-scoring segment pairs (HSPs) in a hit, and 20 (or more, if of the same* E*-value) hits for a query. The transcripts were then annotated using Blast2GO software.

The length of a contig is supposedly the critical factor for successful annotation [40]. Only about 30% of sequences less than 1,000 bp long in our “Nodules” assembly were successfully annotated. The efficiency was 56% for sequences ranging from 1,000 to 2,000 bp. Almost all sequences (93%) longer than 2,000 bp were successfully annotated.

**Figure 4:**
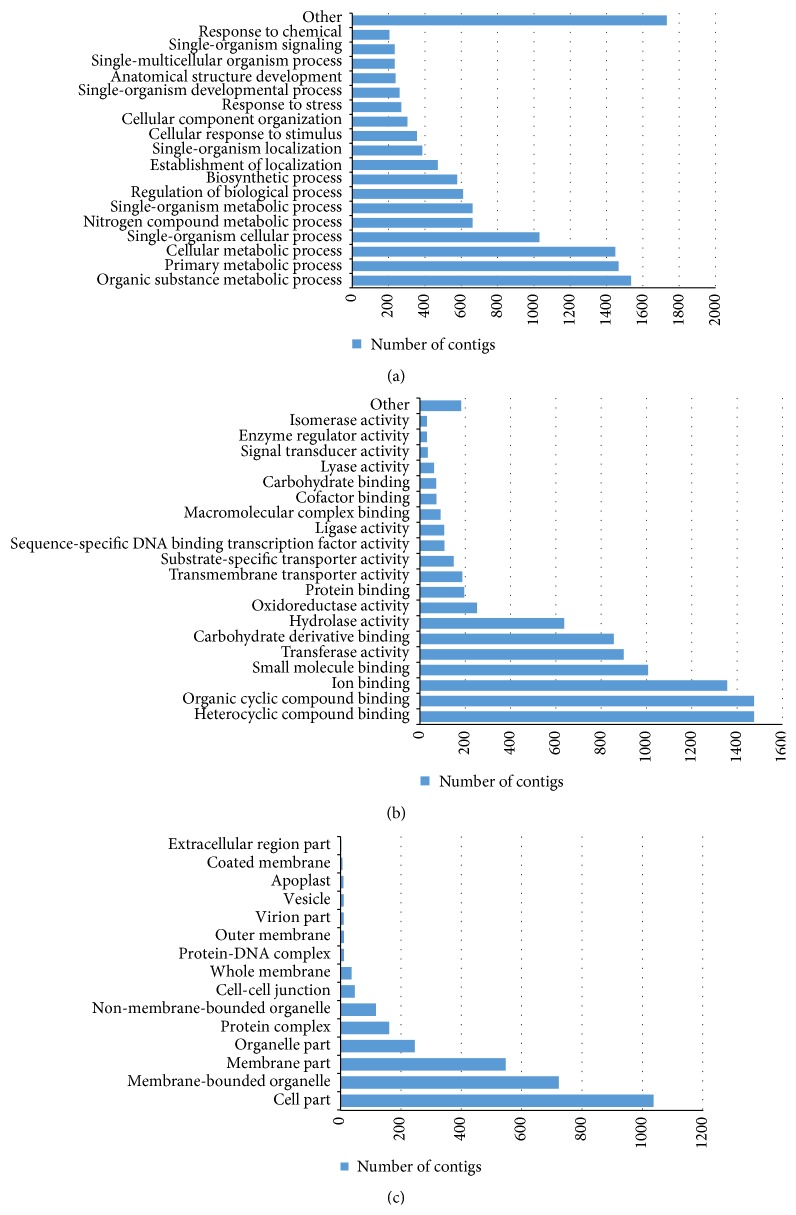
Gene Ontology (GO) classification of nodule-specific contigs. GO subcategories of (a) “biological process,” (b) “molecular function,” and (c) “cellular component” are shown.

One-third of nodule-specific contigs (5,940) were associated with plant proteins in the nr database, with 565,464 total hits. Of these, 3,516 contigs were assigned to 13,697 GO terms. Among biological processes, the most abundant terms were metabolic processes (“organic substance metabolic process”, “primary metabolic process”, “cellular metabolic process”, “single-organism metabolic process”, and “nitrogen compound metabolic process”) along with “single-organism cellular process”, “biosynthetic process”, “establishment of localization”, and “single-organism localization” (Figure 4(a)). This distribution reflects the processes occurring in nodules, such as microsymbiont (rhizobia) hosting within cells and nitrogen compound metabolism. Within the molecular function category, contigs were assigned to “heterocyclic compound binding”, “organic cyclic compound binding”, “ion binding”, “small molecule binding”, “transferase activity”, and “carbohydrate derivative binding” (Figure 4(b)). These terms may be related to metabolite exchange between plants and bacteria, including exchanges with signal molecules. Regarding cellular components, the major GO terms were “cell part”, “membrane-bounded organelle”, “membrane part”, and “organelle part” (Figure 4(c)), which are similarly concerned with the formation and functioning of symbiotic compartments in nodule cells.

It seems also valuable to distinguish the transcripts that are preferentially expressed in nodules as compared to root tips. By mapping the reads of both libraries (“Nodules” and “Root Tips”) to “Nodules” assembly via Bowtie2 v. 2.2.5 and calculating the differential expression via EdgeR package with 0.001 FDR cutoff we selected 1081 contigs that represent genes with significantly higher expression level in nodules (Supplementary File 1 in Supplementary Material available online at http://dx.doi.org/10.1155/2015/695947). Still, more detailed analysis, including verification of the results of such “digital expression” analysis by real-time PCR, is needed, along with addition of more time points to the experiment.

### 3.4. Sequences of Nodule-Specific Transcripts

To identify sequences of novel unreported, highly reliable transcripts of pea, we analyzed the 14,998 sequences of the “nodule only” clusters using TransDecoder software [23]. As a result, 593 putative full-length ORFs were found in 536 contigs (Supplementary File 2).

As an alternative approach to the identification of full-length transcripts, we aligned the same set of 14,998 sequences of the “nodule only” clusters against plant RNA genes in the NCBI RefSeqGene database. Of these sequences, 9,931 had no significant matches. The remaining sequences were filtered according to the following criteria: minimal query coverage of 0.8, maximum* E*-value of 1 × 10^−10^, and the same direction for all HSPs. This step yielded 3,673 contigs and 21,389 hits.

We aligned each pair using the Smith-Waterman algorithm [30] with a “5-0” substitution matrix and identified aligned fragments corresponding to the CDS regions of hit sequences (as determined by GenBank). We selected 427 alignments (comprising 153 unique pea sequences, some of which aligned to multiple GenBank accessions representing gene isoforms or paralogs) with the following characteristics: (1) full coverage of the hit CDS region by a contig; (2) identity higher than 0.8; and (3) the contig having possible start and stop codons within a 50 bp region. Of these 153 contigs, 45 were not detected by TransDecoder.

In total, we identified 581 novel sequences containing putative full-length CDS in pea nodule transcriptome, among which 536 were found by TransDecoder and additional 45 were detected by alternative approach based on BLAST against known plant mRNA sequences. For annotation of these 581 sequences, homologous genes were found by BLASTN search in* Medicago truncatula* genome (ver. 4.0) [45] (see Supplementary File 2). Also, KO (KEGG Orthology) identifiers were assigned to the novel sequences, and 109 entries out of 581 (18.8%) were successfully annotated (Supplementary File 2).

In our opinion, our generated “Nodules” assembly adds valuable information, especially with respect to nodule-specific sequences, to the existing knowledge about pea transcriptome: some unique sequences of pea symbiosis-related genes can be identified only in our assembly. An example of this case involves* CLE* genes, some of which were shown to participate in systemic regulation of nodule formation in several legumes such as* M. truncatula*,* L. japonicus*, and* Glycine max* [46–48]. The* CLAVATA3/Embryo Surrounding Region-Related* (*CLE*) gene family is composed of numerous genes that contain conserved CLE domains in various plant species and encode short regulatory peptides (CLE-peptides) (for review see [49]). In* M. truncatula*, two* CLE* genes,* MtCLE12* and* MtCLE13*, have nodulation-related expression patterns that are linked to proliferation and differentiation [46]. In pea, sequences of CLE genes are not known, but it was shown that overexpression of* MtCLE13* gene leads to similar effects (severe reduction in nodulation) in both pea and* M. truncatula*, proving that* MtCLE13* is functional in pea [50]. So we sought for the sequences homologous to* MtCLE12* and* MtCLE13* (Medtr4g079630.1 and Medtr4g079610.1, resp.) in our nodule transcriptome assembly.

BLASTN search using the Medtr4g079610.1 transcript sequence (encoding Cle13 peptide [46]) as a query against our “Nodules” assembly retrieved the contig TR8317|c0_g1_i1, which contains a full open reading frame (ORF) corresponding to Cle13 of* P. sativum*; the same search against “Organism* Pisum sativum* (taxid: 3880)” in the NCBI TSA database returned two partial transcripts: (1) gb|GCMK01019899.1| (TSA: “Pisum sativum Ps_029064 transcribed RNA sequence”), containing only part of the* Cle13* ORF, and (2) the apparently chimeric gb|GCMO01040960.1| (TSA: “Pisum sativum Ps_150017 transcribed RNA sequence”) containing a portion of the ankyrin repeat gene (similar to* Glycine max* ankyrin repeat-containing protein At5g02620-like [LOC100812799]) as well as the 5′-part of the* Cle13* transcript. A BLASTN search using the Medtr4g079630.1 transcript sequence (encoding Cle12 peptide [46]) as a query found two contigs: TR116|c0_g1_i1, containing the full ORF of* P. sativum Cle12*, and TR23484|c0_g1_i1, containing the full ORF of an unknown protein similar to* P. sativum* and* M. truncatula Cle12* and therefore presumably a paralog of* P. sativum Cle12*. We thus tentatively designated these genes as* Cle12a* and* Cle12b*, respectively (Table 3). At the same time, the search against the NCBI TSA database retrieved no significant homologs of* Cle12* in pea.

The pea transcriptome assembled after Illumina sequencing is thus a good resource for the study of pea transcripts related to nodulation. It can be used in future investigations focused on pea symbiosis-specific genes. Such potential research targets include genes encoding nodule-specific peptides such as NCR- and glycine-rich protein peptides that have been exhaustively described in* M. truncatula* [38, 51] but not* P. sativum*, as well as other symbiotic genes expressed in nodules, including Cle peptide-encoding genes.

Also, the present “Nodules” assembly is a convenient tool that can facilitate study of transcription changes in nodules of symbiotic mutants. In pea, several mutant lines with impaired nodule formation were obtained and phenotypically characterized [52–54]. RNA sequencing of the whole transcriptome from mutant nodules is considered to be a reasonable approach for further characterization of genes and gene networks that operate during nodule development. In this regard, the present assembly of pea nodule transcriptome can be used as a reference for mapping reads and differential expression analysis. Also, this reference transcriptome is indispensable for annotation of short contigs obtained according to the MACE (Massive Analysis of cDNA Ends) protocol, which implies sequencing of 3′-part of each transcript instead of the whole mRNA [55]. Direct annotation of 3′-parts of transcripts by Gene Ontology or KEGG is often not successful because of dissimilarity of these regions between different species, and the present assembly containing significant number of pea nodule-specific transcripts can therefore serve as a reference for annotation of differentially expressed transcripts revealed by MACE technology.

## 4. Conclusions

The aim of the present study was the acquisition of nodule-specific transcript sequences* via* next-generation sequencing. Using an Illumina platform, we obtained 52 million reads from a sample derived from young pea nodules and more than 17 million reads from a root tip sample. We constructed the assemblies (more than 58,000 and 37,000 contigs from nodules and root tips, resp.) and analyzed the “Nodules” assembly for unique sequences. We identified approximately 15,000 nodule-specific contigs associated with different GO biological function terms. Of these, 581 sequences were found to possess full CDSs and could thus be considered as new nodule-specific transcripts of pea.

Because the ability of pea plants to form symbiotic nodules is an agronomically important trait, information about pea nodule-specific gene sequences can be applied by scientists and breeders for primer design, gene-based marker creation, polymorphism studies, and real-time PCR. These findings will thus benefit both fundamental and applied science. The next challenge for researchers is characterization of pea transcripts specific to another symbiosis formed by leguminous plants: arbuscular mycorrhiza, which is also of great importance to both fundamental science and contemporary sustainable agriculture.

## Supplementary Material

Supplementary File 1: Contigs representing genes with significantly higher expression level in nodules that were selected by comparison of “Nodules” and “Root Tips” libraries.
Supplementary File 2: Novel pea nodule-specific sequences containing putative full-length CDSs annotated by: 1) comparison with known plant full-length CDSs (GenBank) and *M. truncatula* genic sequences, and 2) KEGG Orthology mapping.

## Figures and Tables

**Table 1:** Bioprojects from http://www.ncbi.nlm.nih.gov/ containing pea nodule or root tip transcriptome data.

Bioproject	Biosample	SRA	Platform	Submitted by	Description			TSA
					Pea genotype	Sample	Time of harvesting	
PRJNA257308	SAMN02950507	SRX669192	Illumina HiSeq 2000	University of Minnesota, Nevin Young; 2014-08-01	Little Marvel	Nodules	30 days after inoculation	NA
		SRX669212	Illumina HiSeq 2000		Little Marvel	Nodules	30 days after inoculation	NA

PRJNA267198	SAMN03153588	SRS747845	NA		Cameor	Nodules	Development stage, 5-6 open leaves/7-8 nodes (nodules stage A)	NA
	SAMN03153589	SRS752085	NA	INRA, Jonathan Kreplak; 2014-10-30	Cameor	Nodules	Development stage, flowering (nodules stage B)	NA
	SAMN03153591	SRS752086	NA		Cameor	Nodules	Development stage, 18 days after sowing, that is, 10 days after inoculation (nodules stage G)	NA

PRJNA277074	SAMN03396628	SRX952469	Illumina HiSeq 2000		Kaspa	Nodules	3-month-old plants	GCMF00000000.1; GCMG00000000.1; GCMH00000000.1; GCMI00000000.1; GCMJ00000000.1; GCMK00000000.1; GCML00000000.1
		SRX952470	Illumina MiSeq		Kaspa	Nodules	3-month-old plants	
	SAMN03396630	SRX952472	Illumina HiSeq 2000	Department of Economic Development, Jobs, Transport and Resources, 5 Ring Road, Bundoora, VIC 3083, Australia; Shimna Sudheesh; 2015-03-09	Kaspa	Root tips	4-week-old plants	
		SRX952473	Illumina MiSeq		Kaspa	Root tips	4-week-old plants	
PRJNA277076	SAMN03396658	SRX952517	Illumina HiSeq 2000		Parafield	Nodules	3-month-old plant	GCKA00000000.1; GCMM00000000.1; GCMN00000000.1; GCMO00000000.1; GCMP00000000.1; GCMQ00000000.1
		SRX952518	Illumina MiSeq		Parafield	Nodules	3-month-old plant	
	SAMN03396660	SRX952521	Illumina HiSeq 2000		Parafield	Root tips	4-week-old plants	
		SRX952522	Illumina MiSeq		Parafield	Root tips	4-week-old plants	

PRJNA284856	SAMN03733514	SRS945123	Illumina GAIIx	All-Russia Research Institute for Agricultural Microbiology, Saint Petersburg, Russia	SGE	Nodules	12 days	Registered as SUB965211
	SAMN03733554	SRS945125	Illumina GAIIx		SGE	Root tips	12 days	Registered as SUB965299

**Table 2:** Pea nodule transcripts corresponding to known symbiotic genes of *Medicago truncatula* and *Lotus japonicus.*

Gene	Accession number (*M. truncatula* or *L. japonicus*)	Contig in “Nodules”	Forward primer, 5′-3′	Reverse primer, 5′-3′	Identity % (CDS)
*EFD*	EU251063.1	TR2716|c0_g1_i1	ACCTTCACTTCACTTCACTTAAG	GGTGTCATGGAGAAATGCTACA	88%
*NSP1*	AJ972478.1	TR20452|c0_g1_i2	AATGATCCAAGAACACTACTAACC	CAGCTCTCTTAATCACAGACAT	88%
*VPY*	GQ423209.1	TR12524|c0_g2_i1	ACCATCATAAACCAAACTGTTGC	TCCAAATCACACTCACAACTCC	93%
*Sen1*	AB573230.1	TR29546|c0_g1_i1	TAAACAGATCAATCAAGCATTCATG	ATTGGTTCAACATGAAGTATACG	76%^*∗*^

^*∗*^% identity to the *L. japonicus* sequence; for others, % identity to *M. truncatula* sequences is given.

**Table 3:** Pea transcripts homologous to *Medicago truncatula* transcripts encoding Cle12 and Cle13 peptides.

	*M. truncatula* gene	Contig in “Nodules”	Identity % (CDS/protein)	Suggested name for pea transcript
Cle12	Medtr4g079630.1	TR116|c0_g1_i1	79/65	Cle12a
		TR23484|c0_g1_i1	74/50	Cle12b
Cle13	Medtr4g079610.1	TR8317|c0_g1_i1	80/68	Cle13
